# Molecular subtypes, prognostic factors, and treatment optimization in pediatric medulloblastoma: a real-world study from China

**DOI:** 10.3389/fonc.2025.1597123

**Published:** 2025-07-30

**Authors:** Jingjing Liu, Yanling Sun, Huming Li, Shuxu Du, Jin Zhang, Zhen Zhang, Liming Sun, Wanshui Wu

**Affiliations:** ^1^ Department of Pediatrics, Beijing Shijitan Hospital, Capital Medical University, Beijing, China; ^2^ Department of Pulmonary and Critical Care Medicine, 6th Medical Center of Chinese People’s Liberation Army (PLA) General Hospital, Beijing, China

**Keywords:** medulloblastoma, risk factors for relapse and mortality, maintain chemotherapy by risk stratification, large cohort, real-world study

## Abstract

**Background:**

Medulloblastoma (MB) is the most common malignant pediatric brain tumor, yet systematic studies on molecular characteristics and treatment efficacy in Chinese pediatric MB remain scarce. This study evaluates recurrence and mortality risk factors and the feasibility of intensified chemotherapy.

**Methods:**

A retrospective analysis of 381 MB patients (WNT: 63, SHH: 106, Group 3: 27, Group 4: 185) was conducted. Kaplan-Meier analysis estimated survival rates, and Cox regression identified independent risk factors for recurrence and mortality.

**Results:**

With a median follow-up of 4.8 years, 5-year PFS and OS were 69.9% ± 2.4% and 80.6% ± 2.1%, respectively. WNT-MB had the best prognosis, while Group 3-MB had the worst. Independent recurrence risk factors included high-risk status (HR=2.931, *p*<0.001), TP53 mutation (HR=1.873, *p*<0.001), MYCN amplification (HR=1.52, *p*=0.001), chromosome 12p amplification, and 9q deletion. Mortality was associated with LC/A pathology (HR=1.573, *p*=0.007), TP53 mutation (HR=2.049, *p*<0.001), and high-risk status (HR=3.966, *p*<0.001). TP53 mutations influenced WNT-MB recurrence, and Group 3-MB showed a high recurrence risk even without MYC amplification or metastasis. No treatment-related fatalities were observed.

**Conclusion:**

This study identified key clinical and molecular risk factors associated with recurrence and mortality in pediatric medulloblastoma. The findings underscore the prognostic relevance of TP53 mutations, MYCN amplification, and specific chromosomal alterations, particularly in non-metastatic subgroups. These insights may help guide risk-adapted and personalized treatment strategies in future studies.

## 1 Introduction

Medulloblastoma (MB) is an aggressive primary malignancy of the central nervous system (CNS), primarily arising in the cerebellum or dorsal brainstem. It accounts for approximately 20% of pediatric CNS tumors, with peak incidence occurring between 6 and 8 years of age (1). Despite significant advancements in multimodal therapies—including surgical resection, craniospinal irradiation (CSI), and multi-agent chemotherapy—long-term survival beyond five years remains at only 60–80% (2). Tumour recurrence remains a significant challenge, with nearly one-third of patients succumbing to the disease within five years and a median post-relapse survival of approximately one year (3).

Advancements in pathology and molecular biology have led to the classification of MB into four histopathological subtypes—classic (CMB), desmoplastic/nodular (DMB), extensive nodularity (MBEN), and large cell/anaplastic (LC/A) (4, 5)—as well as four molecular subgroups: Wingless activated (WNT). Sonic Hedgehog activated (SHH), non-WNT/non-SHH (include Group 3 and Group 4) (6). These subgroups exhibit distinct biological, clinical, and prognostic profiles. Prior studies report a 5-year progression-free survival (PFS) rate exceeding 90% for the WNT subgroup, whereas Group 3 has the poorest prognosis, with 5-year PFS rates below 50%. SHH and Group 4 demonstrate intermediate outcomes, with 5-year PFS rates ranging from 70% to 75% (1, 6, 7). Although molecular stratification has improved risk assessment and facilitated tailored treatment approaches, the application of targeted therapies remains limited, and most patients continue to rely on conventional treatment modalities.

With advancements in clinical research, risk-stratified treatment approaches for MB have been widely adopted. This approach categorizes patients based on postoperative tumor residual volume and metastatic status, allowing for tailored radiotherapy and chemotherapy regimens (8–10). Risk-stratified treatment of MB helps mitigate long-term cognitive impairment and hearing loss caused by radiotherapy, improve patients’ quality of life, and reduce unnecessary treatment-related injuries (11). Average-risk patients receive lower-dose craniospinal irradiation (CSI, 23.4 Gy) and focal radiotherapy (54 Gy). In contrast, high-risk patients undergo intensified CSI (36–39.6 Gy) combined with more aggressive chemotherapy regimens to improve survival outcomes (12, 13). Well-established risk factors for MB recurrence include TP53 mutations in the SHH subgroup and metastatic disease at diagnosis. However, controversy remains regarding specific patient subgroups, such as WNT MB with TP53 mutations and Group 3 MB without MYC amplification, as their association with recurrence risk has yet to be conclusively determined. Further research is needed to clarify their prognostic significance (2).

Despite these advancements, the long-term outcomes of Chinese pediatric MB patients remain unclear. This study utilizes real-world data from Chinese pediatric MB patients to assess long-term survival and prognostic factors across molecular subgroups (WNT, SHH, Group 3, and Group 4). By analyzing molecular features and survival rates, this research aims to provide a theoretical foundation for precision medicine in Chinese pediatric MB patients, supplement global MB treatment data, bridge the gap in long-term follow-up data for Chinese pediatric MB patients, and explore the association between molecular biomarkers and clinical outcomes. These findings will contribute to optimizing region-specific treatment strategies.

## 2 Methods

### 2.1 Data collection

A retrospective analysis was conducted on pediatric MB patients treated at Beijing Shijitan Hospital, Capital Medical University, between January 2012 and June 2022. Data collected included demographic information, clinical characteristics, and treatment regimens. The Ethics Committee of Beijing Shijitan Hospital, Capital Medical University, approved this study. As a retrospective study using fully de-identified data, it involved no therapeutic interventions and did not affect patient outcomes; therefore, informed consent was waived.

### 2.2 Inclusion and exclusion criteria

Patients were included if they met the following criteria:(1) aged 3 to <18 years; (2) had a histologically confirmed MB diagnosis with defined pathological and molecular subtypes; (3) received postoperative treatment at our institution. Exclusion criteria included (1) tumor recurrence before admission, (2) concurrent malignancies or systemic diseases, and (3) loss of follow-up.

All patients underwent tumor resection and were classified based on postoperative imaging as follows: gross total resection (GTR), near-total resection (NTR, residual tumor ≤ 1.5 cm^2^), and subtotal resection (STR, residual tumor> 1.5 cm^2^).

### 2.3 Pathological classification, molecular subtyping, and risk stratifying

In this retrospective study, MB cases treated before 2016 were classified according to the WHO 2007 classification system5. Cases from 2017 onward were classified based on the WHO 2016 criteria15, which integrate pathological and molecular subtyping. Pathologically, MB was categorized into three major subtypes: CMB, DMB, and LC/A. Due to its significant histological overlap, MBEN was grouped with DMB.

Molecular subtyping stratified MB into four subgroups: WNT, SHH, Group 3, and Group 4. These molecular subgroups exhibit distinct genetic profiles and clinical outcomes, offering valuable insights into MB heterogeneity and prognosis.

Average-risk MB: No residual tumor (postoperative residual volume <1.5 cm²) and M0 disease.

High-risk MB: Presence of residual tumor (≥1.5 cm²) and/or disseminated disease (M1–M3).

New low-risk was defined as the absence of all adverse prognostic factors, while “new high-risk” indicated the presence of at least one.

### 2.4 Treatment: this study adopted the following protocol as the foundation for treatment

#### 2.4.1 Average-risk group

Radiotherapy commenced 3–4 weeks post-surgery, including craniospinal irradiation (CSI, 23.4–30.6 Gy) and a primary boost (PB, 54–55.8 Gy). Maintenance chemotherapy started six weeks after radiotherapy, consisting of 6–8 cycles of cisplatin (70 mg/m^2^, d1), vincristine (1.5 mg/m^2^, max 2 mg, d1,8,15), and lomustine (75 mg/m^2^, d1) (14), or six cycles of this regimen combined with two cycles of cyclophosphamide (1 g/m², d1–2) and vincristine (1.5 mg/m^2^, max 2 mg, d1) (15). For patients unable to tolerate immediate radiotherapy due to complications (e.g., cerebellar mutism), induction chemotherapy with cyclophosphamide/vincristine and carboplatin/etoposide was administered until the patient stabilized for radiotherapy.

#### 2.4.2 High-risk group

There were two pre-radiotherapy cycles, each including cyclophosphamide (800 mg/m^2^, d1–3)/vincristine (1.5 mg/m^2^, max 2 mg, d1), methotrexate (5 g/m^2^, 24-hour infusion)/vincristine (1.5 mg/m^2^, max 2 mg, d1), and carboplatin (200 mg/m^2^, d1–3)/etoposide (100 mg/m^2^, d1–3) (16). Induction chemotherapy began 3–4 weeks post-surgery, consisting of two pre-radiotherapy cycles, each including cyclophosphamide (800 mg/m^2^, d1–3)/vincristine (1.5 mg/m^2^, max 2 mg, d1), methotrexate (5 g/m^2^, 24-hour infusion)/vincristine (1.5 mg/m^2^, max 2 mg, d1), and carboplatin (200 mg/m^2^, d1–3)/etoposide (100 mg/m^2^, d1–3) (16), for patients who received adequate induction chemotherapy. Those without sufficient induction chemotherapy received a more intensive regimen, incorporating cyclophosphamide/vincristine and carboplatin/etoposide, totaling 8–10 cycles of maintenance chemotherapy.

According to the Common Terminology Criteria for Adverse Events (CTCAE) versions 3.0–5.0, grade 1–2 adverse events were classified as mild, while grade 3–4 adverse events were classified as severe in evaluating chemotherapy-related toxicities.

### 2.5 Statistical methods

Continuous variables were expressed as mean ± standard error (SE) or median (interquartile range, IQR). Survival analysis was performed using the Kaplan-Meier method to estimate survival curves, with differences between groups assessed using the log-rank test. Risk factor analysis was conducted using univariate Cox or multivariate Cox proportional hazards regression. All statistical analyses were performed using R software (version 4.3). A p-value < 0.05 was considered statistically significant.

## 3 Results

### 3.1 Patient enrollment and treatment overview

**Figure 1:**
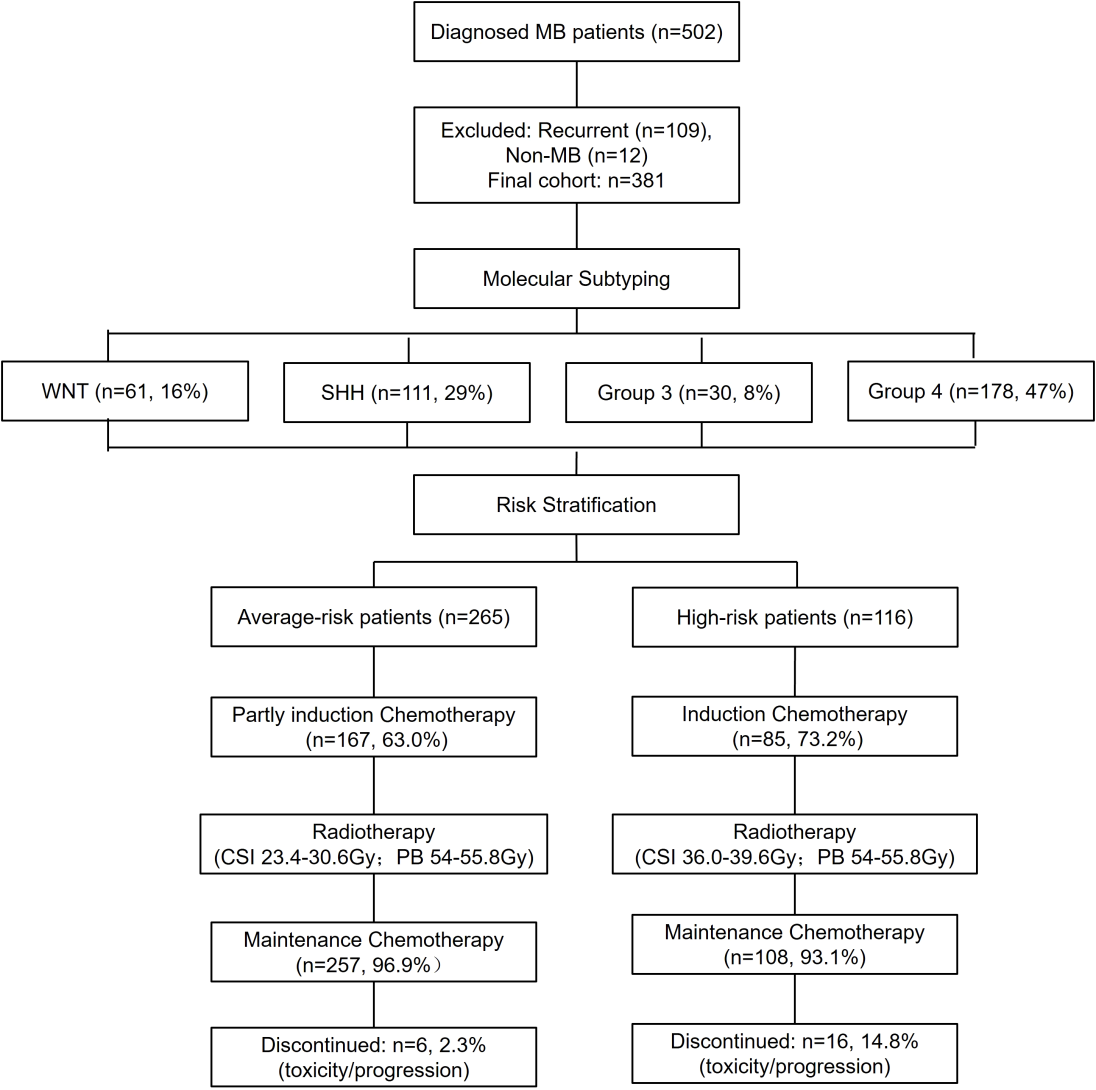
Study flowchart. This flowchart provides a comprehensive overview of patient enrollment, exclusion criteria, and molecular classification. Additionally, it illustrates the distribution of patients into average- and high-risk categories and presents the chemotherapy completion rates. WNT, Wingless activated. SHH, Sonic Hedgehog activated.

A total of 381 MB patients were included after excluding recurrent cases (n=109) and non-MB tumors (n=12), with four molecular subtypes: WNT (16%), SHH (29%), Group 3 (8%), and Group 4 (47%), with an average-risk to a high-risk ratio of 253:128. In this cohort, 20 pediatric patients were diagnosed before 2017, as routine molecular subtyping was implemented at our center only after 2017. Six patients discontinued chemotherapy in the average-risk group (n=265) due to disease progression or adverse effects. In the high-risk group (n=116), 16 patients discontinued chemotherapy due to disease progression or toxicity. (**Figure 1**).

### 3.2 General patient characteristics

**Figure 2:**
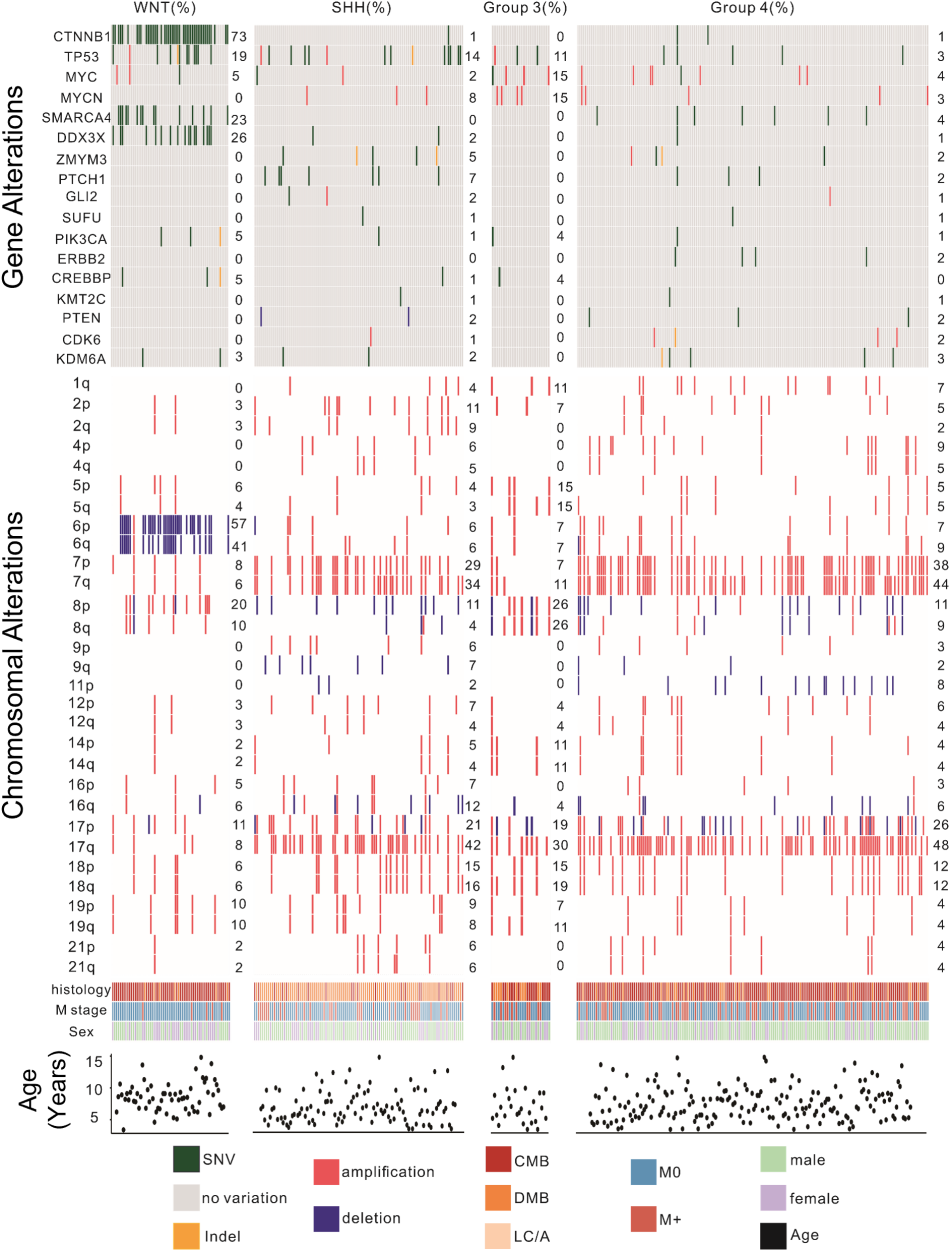
Genomic landscape of pediatric patients with medulloblastoma. This figure illustrates the distribution of significant gene mutations and chromosomal variations among the four molecular subtypes of MB. Key findings include the high prevalence of CTNNB1 mutations and chromosome 6 deletions in the WNT subtype; TP53 mutations and chromosome 7 and 17 amplifications in the SHH subtype; MYC and MYCN amplifications in the Group 3 subtype; MYC, SMARCA4, and MYCN mutations along with chromosome 7 and 17 amplifications in the Group 4 subtype. Genomic alterations were identified via next-generation sequencing or combined with RNA-seq. CMB, classical MB; DMB, desmoplastic nodular MB; LC/A, large cell or anaplastic; SHH, Sonic Hedgehog activated; WNT, Wingless activated. SNV, Single Nucleotide Variant; M0, no evidence for metastatic; M+, metastatic spread of disease.

The majority of patients were male and diagnosed before the age of 12. Group 3 patients had the highest proportion of disseminated metastasis at diagnosis (37%), followed by Group 4 (36.8%), SHH (30.2%), and WNT (7.9%). Pathological classification showed that CMB was the predominant subtype in the WNT and Group 4 subgroups, whereas DMB was most prevalent in the SHH subgroup. Group 3 patients were most frequently diagnosed with CMB and LC/A. (**Table 1**). The Gene Mutations and Chromosomal Variations Across Groups are demonstrated in **Figure 2**. Baseline characteristics, including sex, histopathological subtype, M-stage distribution, and risk stratification, were well balanced across the four groups, with no statistically significant differences observed, except for the extent of surgical resection.

**Table 1:** Demographic and baseline characteristics of the patients.

Characteristic	ALL	WNT [n (%)]	SHH [n (%)]	Group3 [n (%)]	Group4 [n (%)]	*P* value
	n=381	n=63	n=106	n=27	n=185	
Sex						
male	253	32 (50.8%)	71 (67.0%)	16 (59.3%)	134 (72.4%)	0.014
female	128	31 (49.2%)	35 (33.0%)	11 (40.7%)	51 (27.6%)	
Extent of resection						
GTR	287	54 (85.7%)	78 (73.6%)	20 (74.1%)	135 (73.0%)	0.562
NTR	86	8 (12.7%)	26 (24.5%)	6 (22.2%)	46 (24.8%)	
STR	8	1 (1.6%)	2 (1.9%)	1 (3.7%)	4 (2.2%)	
M stage						
M0	266	58 (92.1%)	74 (69.8%)	17 (63%)	117 (63.2%)	<0.001
M+	115	5 (7.9%)	32 (30.2%)	10 (37%)	68 (36.8%)	
Histology						
CMB	246	56 (88.9%)	22 (20.8%)	16 (59.3%)	152 (82.2%)	<0.001
DMB	104	6 (9.5%)	78 (73.5%)	3 (11.1%)	17 (9.2%)	
LC/A	31	1 (1.6%)	6 (5.7%)	8 (29.6%)	16 (8.6%)	
Risk						
Average risk	265	58 (92.1%)	73 (68.9%)	17 (63.0%)	117 (63.2%)	<0.001
High risk	116	5 (7.9%)	33 (31.1%)	10 (37.0%)	68 (36.8%)	

This table presents the demographic and clinical characteristics of patients categorized by molecular subtypes.

GTR, gross resected; NTR, near totally resected; STR, subtotally resected; M+, metastatic spread of disease; M0, no evidence of metastatic disease; CMB, classical MB; DMB, desmoplastic nodular MB; LCA, large cell anaplastic MB; WNT, Wingless activated. SHH, Sonic Hedgehog activated; CT, chemotherapy; RT, radiotherapy.

### 3.3 Overall survival and progression-free survival

**Figure 3:**
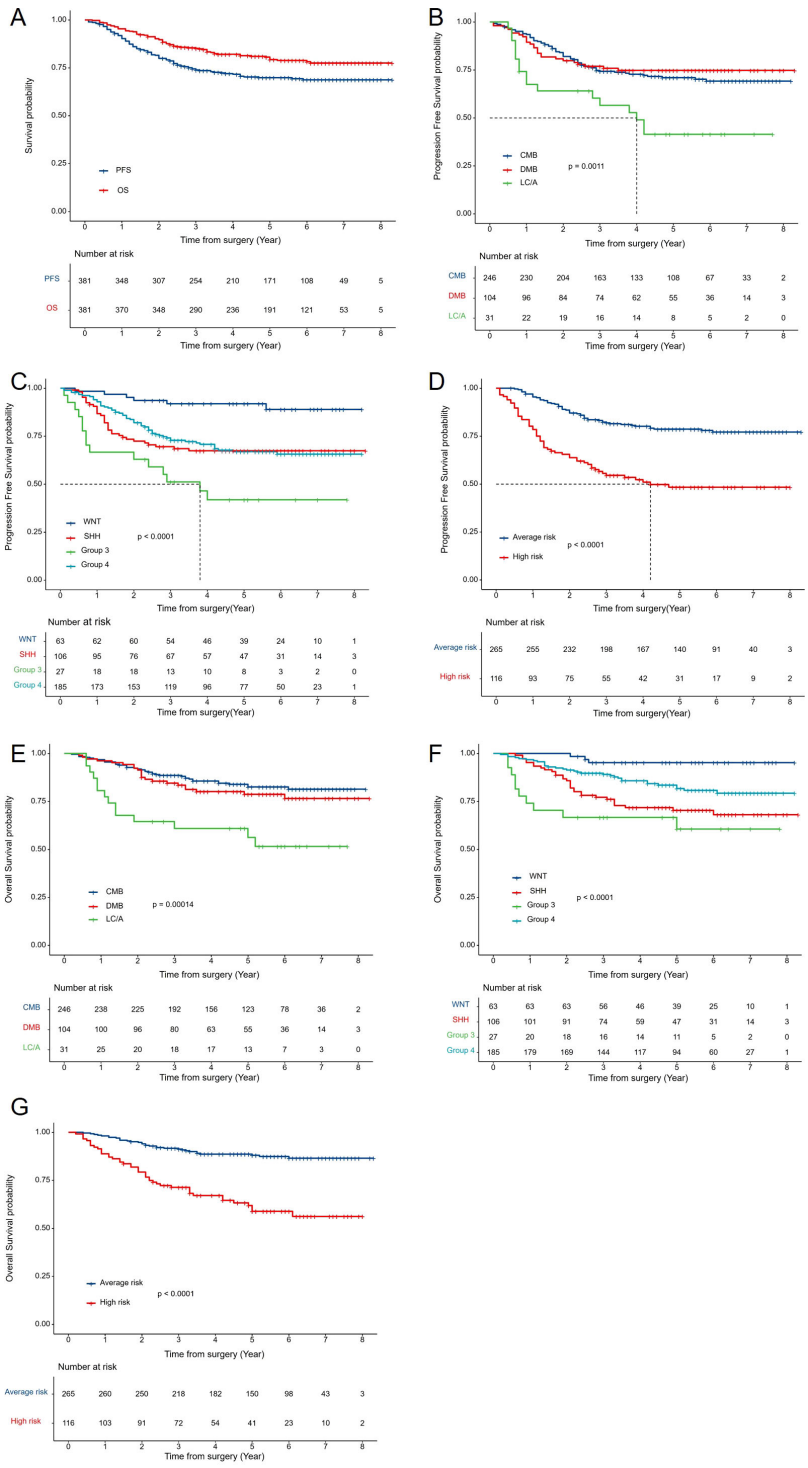
Survival Analysis by Clinical and Molecular Subgroups: **(A)** Kaplan-Meier curves for 5-year PFS and OS of all patients with MB. **(B)** PFS by histological subtype (CMB, DMB, LC/A). **(C)** PFS by molecular subtype (WNT, SHH, Group 3, Group 4). **(D)** PFS by risk stratification (average-risk vs. high-risk groups). **(E)** OS by histological subtype. **(F)** OS by molecular subtype. **(G)** OS by risk stratification. OS, overall survival; PFS, progression-free survival.AR, average risk; HR, high risk.CMB, classical MB; DMB, desmoplastic nodular MB; LC/A, large cell or anaplastic; SHH, Sonic Hedgehog activated; WNT, Wingless activated.

The median follow-up was 4.8 years (range 0.2–8.3 years). The 5-year PFS and OS rates were 69.9% ± 2.4% and 80.6% ± 2.1%, respectively (**Figure 3A**). By risk group, the 5-year PFS was 78.7% ± 2.6% for the average-risk group and 58.8% ± 5% for the high-risk group (p<0.0001) (**Figure 3D**). The 5-year PFS for CMB, DMB, and LC/A were 71.3% ± 3%, 74.7% ± 4.3%, and 41.5% ± 9.3%, respectively(p=0.0011) (**Figure 3B**). Among molecular subtypes, the 5-year PFS rates were 91.9% ± 3.5% for WNT, 67.4% ± 4.6% for SHH, 41.9% ± 9.9% for Group 3, and 66.9% ± 3.7% for Group 4 (p<0.0001) (**Figure 3C**). For OS, the 5-year rates were 88% ± 2.1% for the average-risk group and 58.8% ± 5% for the high-risk group (p<0.0001) (**Figure 3G**). The 5-year OS for CMB, DMB, and LC/A were 83.9% ± 2.5%, 78.7% ± 4.2%, and 60.9% ± 8.8%, respectively (p=0.00014) (**Figure 3E**). For molecular subtypes, the 5-year OS rates were 95.2% ± 2.7% for WNT, 70.3% ± 4.6% for SHH, 60.6% ± 10.1% for Group 3, and 81.7% ± 3.1% for Group 4 (p<0.0001) (**Figure 3F**).

### 3.4 Analysis of risk factors for MB recurrence and mortality

COX regression analysis revealed that high-risk status, TP53 mutation, and MYCN amplification were independent risk factors for recurrence. In addition to the commonly reported high-risk status at diagnosis, TP53 mutation, and MYCN amplification, our study identified chromosome 6p deletion as a protective factor against recurrence, consistent with the well-established association of WNT subgroup tumors with chromosome 6 deletion. Interestingly, we found that chromosome 12p amplification and chromosome 9q deletion were both significant risk factors for recurrence, with hazard ratios (HRs) of 1.567 (95% CI: 1.034–2.375) and 3.028 (95% CI: 1.621–5.658), respectively(**Table 2.1**). Similarly, high-risk status at diagnosis, large cell/anaplastic (LC/A) histology, MYCN amplification, and chromosome 12p amplification were identified as independent risk factors for mortality in MB patients. COX regression analysis further indicated that LC/A histology was significantly associated with increased mortality risk, whereas molecular subgrouping did not show a statistically significant association with recurrence risk or mortality risk (**Table 2.2**).

**Table 2.1:** Risk factors of MB recurrence in the cohort by Univariable and multi-COX analyses.

Characteristics	Univariable analyses		Multivariable analyses	
	HR (95% CI)	p value	HR (95% CI)	p value
Risk stratification	3.061(2.115-4.432)	<0.001	2.931(2.01-4.275)	<0.001
TP53 mutation	1.655(1.216-2.253)	0.001	1.873(1.37-2.561)	<0.001
MYCN amplification	1.421(1.106-1.826)	0.006	1.52(1.179-1.961)	0.001
MYC amplification	1.37(1.067-1.76)	0.014		
chr 6p deletion	0.537(0.371-0.883)	0.012	0.573(0.357-0.922)	0.022
chr 9q deletion	1.634(1.083-2.467)	0.019	1.567(1.034-2.375)	0.034
chr12p amplification	2.617(1.437-4.766)	0.002	3.028(1.621-5.658)	0.001
chr21p amplification	2.321(1.13-4.766)	0.022		

**Table 2.2:** Risk factors of MB mortality in the cohort by Univariable and multi-COX analyses.

Characteristics	Univariable analyses		Multivariable analyses	
	HR (95% CI)	p value	HR (95% CI)	p value
Risk stratification	3.831(2.427-6.047)	<0.001	3.966(2.432-6.468)	<0.001
Histology	1.726(1.265-2.355)	0.001	1.573(1.131-2.189)	0.007
TP53 mutation	1.604(1.127-2.282)	0.009	2.049(1.409-2.98)	<0.001
CTNNB1 mutation	0.255(0.08-0.809)	0.02		
MYCN amplification	1.637(1.269-2.113)	<0.001	1.666(1.27-2.186)	<0.001
MYC amplification	1.405(1.049-1.881)	0.023		
chr 2p amplification	2.189(1.091-4.391)	0.027		
chr 6p deletion	0.542(0.311-0.945)	0.031		
chr 7q amplification	1.576(1.001-2.481)	0.049		
chr 12p amplification	3.382(1.784-6.411)	<0.001	2.614(1.202-5.683)	0.015
chr 21p amplification	2.43(1.055-5.596)	0.037		

HR, hazard ratio; chr, Chromosome.

### 3.5 Recurrence risk factors and new stratification in M0 MB patients

**Figure 4:**
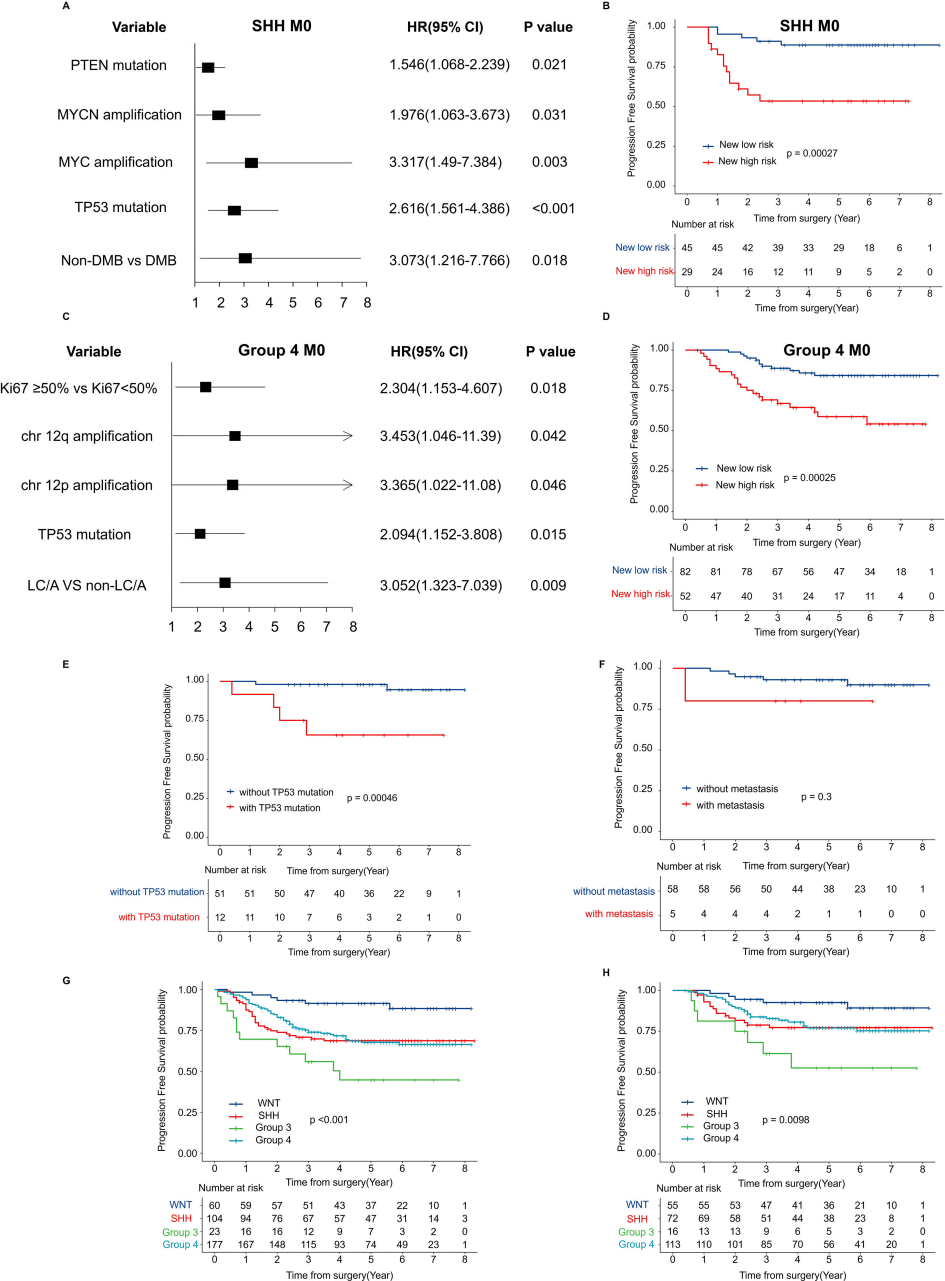
Risk factor modeling and survival analysis by new risk classification **(A)** Forest plot displaying HR and 95% CI for recurrence-associated factors in SHH M0 MB, estimated using univariate Cox regression analysis. **(B)** Kaplan-Meier survival curves for PFS by the proposed new risk classification in SHH M0 MB. **(C)** Forest plot showing HR and 95% CI for recurrence-associated factors in the combined cohort of Group 3 and Group 4 M0 MB, derived from univariate Cox regression analysis. **(D)** Kaplan-Meier PFS estimates stratified by the new risk classification in the combined cohort of Group 3 and Group 4 M0 MB. **(E)** PFS in the WNT subgroup stratified by TP53 mutation status; **(F)** PFS in the WNT subgroup stratified by M stage; **(G)** PFS in the no-MYC amplification subgroup by molecular; **(H)** PFS in the no-MYC amplification and M0 stage subgroup by molecular. HR, hazard ratio; CI, confidence intervals; MB, medulloblastoma; PFS, progression free survival; WNT, Wingless activated.

In addition to the overall survival and multivariate analyses mentioned above, we conducted further investigations into specific subtypes. Among patients in the SHH M0 group, PTEN mutation, MYC amplification, MYCN amplification, TP53 mutation, and non-DMB histology were identified as significant prognostic factors (**Figure 4A**). Based on these factors, a newly proposed risk stratification revealed a significant difference in 5-year PFS between the low-risk and high-risk groups (p < 0.001) (**Figure 4B**). Important prognostic factors for non-WNT/non-SHH M0 patients included Ki67 expression, LC/A, chromosome 12 amplification, and TP53 mutation (**Figure 4C**). The new risk-based stratification also demonstrated significant differences in 5-year PFS (p < 0.001) (**Figure 4D**). Several potential risk factors that remain unclear require further investigation. In the WNT subgroup, the 5-year PFS significantly differed between patients without TP53 mutation and those with TP53 mutation (p = 0.00046) (**Figure 4E**). However, in the WNT subgroup, the presence or absence of metastatic dissemination was not a significant risk factor for recurrence (p = 0.3) (**Figure 4F**). To determine whether the high recurrence risk in Group 3 is primarily driven by MYC amplification or metastatic status, we analyzed patients without MYC amplification. The results indicated that, compared to other subgroups, Group 3 patients exhibited a significantly higher risk of recurrence (**Figure 4G**). Moreover, among MYC-non-amplified patients with M0 staging, Group 3 patients still displayed an elevated recurrence risk (**Figure 4H**).

### 3.6 Treatment-related toxicity

Chemotherapy-related adverse events primarily involve hematologic and non-hematologic toxicities. Hematologic toxicities include neutropenia, anemia, and thrombocytopenia, affecting approximately 40% of patients. Among these, 70% were mild, while 30% were classified as severe. All cases improved with supportive treatments, including granulocyte colony-stimulating factor (G-CSF) supplementation, interleukin-11 (IL-11), and thrombopoietin (TPO) therapy. Non-hematologic toxicities were predominantly gastrointestinal, with common manifestations such as reduced appetite, abdominal pain, and nausea. Some patients also experienced fatigue, which could be attributed to chemotherapy or insufficient nutritional intake. Additionally, chemotherapy-induced immunosuppression increased the risk of infections, primarily affecting the respiratory and gastrointestinal systems. However, all cases were resolved with appropriate anti-infective treatment. No treatment-related mortality was observed in this study (**Table 3**).

**Table 3:** Treatment-related adverse effects in MB patients.

n=381	No. Patients		
Adverse effect	Mild adverse	Severe adverse	Total
Hematological			
Neutropenia	103	48	151
Anemia	78	29	107
Thrombocytopenia	95	38	133
Nonhematological			
Vomiting	63	0	63
Abdominal pain	35	0	35
Fatigue	27	0	27
Oral ulcer	38	2	40
Peripheral neuropathic pain	39	3	42
Nausea	87	3	90
Anorexia	135	0	135
Constipation	113	0	113
Intestinal obstruction	10	0	10
Infection			
Respiratory infection	57	2	59
Intestinal bacterial infection	20	0	20

## 4 Discussion

Medulloblastoma (MB) is a high-grade malignancy of the CNS, which complicates early diagnosis and treatment. First described in 1925, MB remains one of the most challenging pediatric brain tumors due to its heterogeneous clinical presentations and poor prognosis in certain subgroups (17). This study represents one of the most extensive single-center retrospective analyses conducted in China, systematically assessing recurrence, prognosis, and risk factors for recurrence and mortality in Chinese children diagnosed with MB in a real-world setting. Our findings underscore the importance of risk stratification at diagnosis, histopathological subtypes, and molecular classification in predicting prognosis. These results further emphasize the necessity of personalized treatment strategies tailored to individual risk profiles, which may improve clinical outcomes for MB patients in China.

Despite extensive research on MB treatment in high-resource settings, data from China remain limited. A notable study conducted in 2021 by the Chinese Children’s Cancer Group (CCCG) involved 221 MB patients from 12 centers, following the CCCG-MB-2017 guidelines for age- and risk-based treatment stratification (18). Treatment regimens included surgery, radiotherapy, and chemotherapy, primarily consisting of cisplatin, cyclophosphamide, and vincristine. The median follow-up period for this cohort was 2.4 years, with a 3-year PFS rate of 71.5% ± 3.3% and an OS rate of 85.6% ± 2.6%. However, this study’s relatively short follow-up duration contrasts with our long-term follow-up data, which includes patients older than 3 years. This study helps bridge a critical gap in the international literature regarding large-sample, long-term follow-up data on MB in China.

Several clinical trials have been conducted to improve treatment outcomes for children with MB. The HIT2000 protocol is one of the most effective regimens for childhood MB. Bueren et al. (19) applied this protocol to 123 children aged ≥4 years with disseminated MB, achieving a median follow-up of 5.38 years. The 5-year event-free survival (EFS) and OS rates were 62% (95% CI: 52–72%) and 74% (95% CI: 66–82%), respectively, demonstrating a significant improvement over the previous HIT91 protocol (20), which lacked induction chemotherapy and had a 5-year EFS of only 42% (95% CI: 24–60%). The HIT-SIOP PNET4 (21) trial included 340 MB patients, comparing two groups receiving different radiotherapy doses while maintaining the same chemotherapy regimen post-radiotherapy. After a median follow-up of 4.8 years, the 5-year EFS rate was 82% ± 2%. Gandola et al. (22) conducted a study involving 33 MB patients, after a median follow-up period of 82 months, the 5-year EFS, PFS, and OS rates were 70%, 72%, and 73%, respectively. In a study by Jakacki et al. (23), within the Children’s Oncology Group (COG), treatment outcomes for metastatic MB patients were analyzed. The 5-year OS rates ranged from 68% to 82%, while the 5-year PFS rate ranged from 59% to 71%, with no statistically significant difference observed. Similarly, the SJMB03 protocol (23) included 103 high-risk MB patients, reporting a 5-year PFS of 68.1% (± 6.6%), which is comparable to the results from the HIT2000 protocol designed for high-risk patients. The median follow-up for the current study was 4.8 years, revealing a 5-year PFS for the high-risk group of 48.4% (± 4.9%).

Due to the aggressive nature of MB, chemotherapy could be enhanced to help recover from myelosuppression. We adopted a treatment approach combining the SIOP PNET4 and HIT2000 protocols. Intensity-modulated radiotherapy (IMRT) followed by maintenance chemotherapy was utilized to ensure treatment tolerability while reducing the risk of recurrence. In the average-risk group, the 5-year PFS rate was about 75%, and the 5-year OS rate was about 85%. These results align with previous reports, indicating that this cohort’s prognosis for average-risk patients is comparable to international outcomes. This provides valuable therapeutic experience and offers significant clinical implications for improving PFS and OS in MB patients in these areas. However, despite extensive research and exploration, no definitive targeted therapy for MB has been identified, and treatment remains primarily reliant on conventional chemotherapy. Some studies suggest that thyroid hormone T3 may inhibit MB tumor cell proliferation and promote neuronal differentiation (24). This finding suggests a potential new avenue for MB treatment.

Notably, the WNT group, SHH group, and Groups 3 and 4 display unique molecular profiles that guide the development of targeted therapies. These molecular subtypes affect prognosis and open new avenues for personalized treatment strategies (25). Multiple studies have demonstrated that WNT-MB has a favorable prognosis when treated with standard therapies. The majority of WNT-MBs contain CTNNB1 mutations and show 6q chromosome deletions. In our cohort, the mutation rate of CTNNB1 in WNT-MB was 73%, and the deletion rate of the 6q chromosome was approximately 50%. In comparison, previous reports indicate that the CTNNB1 mutation rate was 92%, with a 6q deletion rate of 76%, both of which were higher than those observed in our group, which may be related to ethnic differences. It is worth noting that even within the WNT subtype, reducing the radiotherapy dose to 18 Gy resulted in inferior outcomes (26). Nonetheless, the 5-year PFS rate for this group was significantly higher than that for other subtypes, which aligns with findings from prior studies (27). Even though the WNT group demonstrates better outcomes than other molecular subgroups, our data suggest that TP53 mutation is a high-risk factor for recurrence in the WNT subgroup. However, although the WNT subgroup with dissemination at diagnosis exhibited a trend toward higher recurrence, the difference was not statistically significant. This may be attributed to the limited number of WNT patients presenting with dissemination at diagnosis. Based on analyses of other molecular subtypes, dissemination may still represent a potential high-risk factor for recurrence, even in patients classified within the WNT subgroup. This result is inconsistent with previous reports suggesting that TP53 mutations in the WNT subtype do not impact prognosis (28), and aligns with the recent study (29).

The SHH subtype accounts for approximately one-third of MB cases and exhibits various histological features, including classic, nodular, and extensive nodular types. Tumors in this subtype generally carry mutations in key components of the SHH pathway, such as PTCH1, SMO, Suppressor of Fused Homolog (SUFU), or amplification of the Glioma-associated Oncogene 2 (GLI2). Furthermore, mutations or amplifications in TP53 and MYCN also activate the SHH pathway (30). In our cohort, the most common mutations were found in TP53 (14%), followed by MYCN amplification (8%) and PTCH1 mutations (7%). The most frequently observed chromosomal variations were 17q amplification (42%) and 7q amplification (34%). Notably, GLI2 and SUFU mutations or amplifications were exclusively observed in SHH and Group 4 cases. While other studies have reported PTCH1 as the most frequent mutation gene (47%) and 9q deletion as the most common chromosomal alteration (55%), the gene mutations and chromosomal variations in the SHH subtype in our study exhibit different characteristics, which may be attributed to ethnic differences. The 5-year PFS rate for the SHH group was intermediate, situated between the rates for the WNT group and Group 3, consistent with findings in existing literature.

In Group 3 cases, MYC amplification is a hallmark feature and one of the most common markers associated with poor prognosis (30). MYC-amplified tumors often present with metastasis at diagnosis, contributing to the unfavorable prognosis of this subtype. In our cohort, MYC and MYCN amplifications were most frequently observed in Group 3, with 75% of patients harboring MYC amplification presenting with spinal cord dissemination at diagnosis. Although Group 3 patients frequently exhibit MYC or MYCN amplification, which is often associated with poor prognosis, our study indicates that even in M0 patients without MYC amplification, the PFS of Group 3 remains significantly lower than that of other subgroups. These findings suggest that Group 3 remains a high-risk subgroup for recurrence, even without MYC amplification, underscoring the necessity of high-risk treatment strategies for all Group 3 patients, regardless of MYC status. Group 4 MB lacks distinct genetic markers. Despite the large sample size, no significant mutations were identified in this group, with mutation and amplification rates below 5%. Chromosomal abnormalities, such as amplifications of chromosomes 7 and 17, were commonly observed, but studies, including ours, have shown that these chromosomal alterations do not directly correlate with prognosis (31). Although chromosome 11 deletion may be a protective factor against recurrence in Group 4 patients, our findings showed only a trend toward reduced recurrence, without statistical significance. Further studies are needed to validate this observation.

This study identified several key risk factors for recurrence in patients within the SHH M0 group. Notable factors include non-DMB pathology, TP53 mutation, MYCN amplification, PTEN mutation, and MYC amplification. These findings indicate that SHH-MB patients with DMB pathology tend to have relatively better prognoses, consistent with prior research on the SHH-MB. SHH MB associated with TP53 mutations (SHHα) has been linked to poorer outcomes, while PTEN mutations are similarly correlated with unfavorable prognosis and an increased risk of metastasis (30). In contrast, for patients in the non-WNT, non-SHH M0 group, several significant factors are associated with a higher likelihood of recurrence. These include the LC/A pathological type, TP53 mutations, chromosome 12 amplification, and Ki67 expression levels of 50% or higher. The high recurrence rate observed in the LC/A type is likely due to its aggression. TP53 mutations and the amplification of chromosome 12 may contribute to genomic instability, facilitating rapid tumor cell proliferation and enhancing drug resistance. Additionally, elevated Ki67 expression (≥50%) indicates increased cell proliferation rates, strongly correlating with a heightened risk of recurrence. The risk factors for SHH M0-MB and non-WNT non-SHH M0-MB also differ. Research from the SJMB03 study conducted in Europe showed that the deletion of chromosome 17p, the LC/A type, MYCN amplification, and GLI2 amplification are risk factors for recurrence in M0 SHH-MB. In contrast, MYC amplification is identified as a risk factor for recurrence in M0 Group 4 MB.

This study underscores key molecular markers and pathological features linked to MB recurrence in Chinese children, vital for risk stratification and personalized treatment. Factors associated with poor survival outcomes after recurrence include MYCN amplification, DMB pathology, high-risk status at diagnosis, and older age. Patients with MYC and MYCN amplifications tend to have more aggressive tumors and increased resistance to chemotherapy, leading to worse prognoses. Interestingly, older patients might have a lower mortality risk due to better tolerance for secondary treatments. This study compared PFS between disseminated and non-disseminated patients in the WNT subgroup, revealing no statistically significant difference. However, given the limited sample size (only four disseminated cases), there was a trend toward increased recurrence in disseminated patients. The small sample size may have introduced statistical bias, necessitating further studies with larger cohorts. Clinically, disseminated WNT patients should not be classified as low-risk.

Granulocytopenia or agranulocytosis improved with G-CSF or PG-CSF, while thrombocytopenia was alleviated with IL-11, TPO, or oral TPO receptor agonists (TPO-RAs). However, the optimal timing for TPO-RA use in pediatric patients remains unclear. Mild to moderate anemia responded well to dietary adjustments, while severe cases required red blood cell transfusions to prevent chemotherapy delays. Some patients developed incomplete intestinal obstruction after the first CDDP/VCR/lomustine cycle post-radiotherapy, likely linked to VCR administration. Substituting VCR with VDS (3.0 mg/m²) in the first cycle and continuing VCR (1.5 mg/m²) thereafter prevented recurrence. Peripheral neuropathic pain, possibly due to reduced activity and neurotoxicity, improved with vitamin B12, D3, and calcium supplementation. Analgesics such as ibuprofen were effective for severe cases.

### 4.1 Limitations

This single-center, retrospective analysis provides valuable insights into the treatment and prognosis of Chinese children with MB. However, the findings may not fully represent the broader clinical landscape across diverse institutions in China. The findings presented in this study are primarily hypothesis-generating and exploratory in nature. Therefore, validation in prospective, multi-center settings is essential to enhance the robustness and generalizability of these results. Although methylation profiling was not applied for molecular subgrouping in this study—mainly due to technical limitations in the early part of the cohort—it represents a more accurate classification method. We plan to incorporate methylation-based classification in future studies. Due to limited sample sizes in certain subgroups, such as WNT/TP53-mutant, SHH M0, and Group 3 without MYC amplification, results may be biased and should be interpreted with caution.

### 4.2 Summary

This study represents the first systematic evaluation of long-term treatment efficacy for MB in Chinese children, utilizing a substantial sample of real-world data and addressing a significant gap in the existing literature. Furthermore, the research highlights notable differences in treatment responses and prognostic characteristics between the Chinese population and international cohorts, offering vital insights for future investigations. Integrating molecular biology studies with international collaborations will be essential for optimizing tailored treatment strategies for Chinese children with MB.

## Data Availability

The original contributions presented in the study are included in the article/**Supplementary Material**. Further inquiries can be directed to the corresponding authors.
