# Supplementary material for: Molecular subtypes, prognostic factors, and treatment optimization in pediatric medulloblastoma: a real-world study from China
Source: Front Oncol. 2025 Jul 30;15:1597123. doi: 10.3389/fonc.2025.1597123 (PMC12343238; doi:10.3389/fonc.2025.1597123)
Supplement: Supplementary file 2 [file SupplementaryFile2.docx]

Age grade： 3-6y—1

6-12—2

12-18—3

sex:1 male，2 female

section：0，GTR；1，NTR；3，PTR

HIS：CMB1;DMB2;MBEN3;LC/A4

his combine：1，CMB;2,DMB,3,LCA(DMB和MBEN合并为DMB)

MOL:WNT1;SHH2;G3 3;G4 4；5，NOS

treatment：C 0;RC 1;CRC 2

gene：

0，no change；1，SNV，2， indel; 3, amplification;4, deletion

chr：0 no change，1 amplification，2 deletion

risk:0 average risk;1 high risk

ki67 50%：1，less；2 more than

M01: 0:M0; 1:M+

ki67 risk group：

average risk：ki67 <50%,1; ki67 >50%, 2;

high risk：ki67 <50%,3; ki67 >50%, 4;

risknew:

SHH M0: 0 low risk(A17q，DMB); 1 average risk ; 2 high risk(no-DMB，mycn,tp53,myc,A6Q,A12P)

G3G4 M0: 0, average risk; 1, high risk
